# Competing Interface and Bulk Effect–Driven Magnetoelectric Coupling in Vertically Aligned Nanocomposites

**DOI:** 10.1002/advs.201901000

**Published:** 2019-08-02

**Authors:** Aiping Chen, Yaomin Dai, Ahmad Eshghinejad, Zhen Liu, Zhongchang Wang, John Bowlan, Erik Knall, Leonardo Civale, Judith L. MacManus‐Driscoll, Antoinette J. Taylor, Rohit P. Prasankumar, Turab Lookman, Jiangyu Li, Dmitry Yarotski, Quanxi Jia

**Affiliations:** ^1^ Center for Integrated Nanotechnologies (CINT) Los Alamos National Laboratory Los Alamos NM 87545 USA; ^2^ Department of Mechanical Engineering University of Washington Seattle WA 98195 USA; ^3^ Theoretical Division Los Alamos National Laboratory Los Alamos NM 87545 USA; ^4^ Department of Quantum and Energy Materials International Iberian Nanotechnology Laboratory Braga 4715‐330 Portugal; ^5^ MPA‐CMMS Los Alamos National Laboratory Los Alamos NM 87545 USA; ^6^ Department of Materials Science and Metallurgy University of Cambridge 27 Charles Babbage Rd. Cambridge CB3 OFS UK; ^7^ Department of Materials Design and Innovation University at Buffalo—The State University of New York Buffalo NY 14260 USA

**Keywords:** epitaxial, interfaces, magnetoelectric couplings, nanocomposites, strain

## Abstract

Room‐temperature magnetoelectric (ME) coupling is developed in artificial multilayers and nanocomposites composed of magnetostrictive and electrostrictive materials. While the coupling mechanisms and strengths in multilayers are widely studied, they are largely unexplored in vertically aligned nanocomposites (VANs), even though theory has predicted that VANs exhibit much larger ME coupling coefficients than multilayer structures. Here, strong transverse and longitudinal ME coupling in epitaxial BaTiO_3_:CoFe_2_O_4_ VANs measured by both optical second harmonic generation and piezoresponse force microscopy under magnetic fields is reported. Phase field simulations have shown that the ME coupling strength strongly depends on the vertical interfacial area which is ultimately controlled by pillar size. The ME coupling in VANs is determined by the competition between the vertical interface coupling effect and the bulk volume conservation effect. The revealed mechanisms shed light on the physical insights of vertical interface coupling in VANs in general, which can be applied to a variety of nanocomposites with different functionalities beyond the studied ME coupling effect.

Large magnetoelectric (ME) coefficients have been achieved in bulk multiferroic composites.^1^, ^2^ Recent advances in thin‐film synthesis have enabled the fabrication of multiferroic thin films and nanocomposites for applications in microelectronic devices.^3^, ^4^, ^5^, ^6^, ^7^, ^8^, ^9^ Vertically aligned nanocomposite (VAN) thin films, consisting of two phases epitaxially grown on a substrate, have demonstrated a unique approach for controlling vertical interfacial area and vertical lattice strain.^10^, ^11^ In comparison with single phase ME materials, these nanocomposites allow us to choose constituents with both large piezoelectric and magnetostrictive coefficients. Therefore, nanocomposites could lead to artificial materials with superior multiferroic properties and ME coupling.

Due to the large vertical interface and possibly reduced substrate clamping effect in VAN films compared to lateral heterostructures, VANs are more favorable for ME couplings. Zheng et al. pioneered the synthesis of lead‐free multiferroic BaTiO_3_:CoFe_2_O_4_ (BTO:CFO) VANs.^12^ Nan et al. have predicted that VANs exhibit larger ME coupling coefficients than multilayer structures.^13^ A large room‐temperature ME coupling coefficient of 2 V cm^−1^ Oe^−1^ was theoretically predicted in BTO:CFO nanocomposites.^14^ Furthermore, Schmitz‐Antoniak et al. have used soft X‐ray absorption spectroscopy to understand the existence of ME couplings in BTO:CFO nanocomposites.^15^ The ME coupling effect and multiferroic properties have been explored in other VANs including the BiFeO_3_:CoFe_2_O_4_ and Pb(ZrTi)O_3_:CoFe_2_O_4_ systems.^16^, ^17^, ^18^, ^19^, ^20^, ^21^ The measured direct ME (DME) coupling coefficients are in the range of 8–390 mV cm^−1^ Oe^−1^.

Both DME effect with magnetic field control of ferroelectricity and converse ME (CME) (electric field control of magnetism) have been used to evaluate ME coupling strength. The CME effect has been used to characterize layered heterostructures^22^, ^23^, ^24^, ^25^ and VANs.^18^, ^26^, ^27^ For example, a CME effect was recently reported at room temperature in a Na_0.5_Bi_0.5_TiO_3_:CoFe_2_O_4_ VAN system with low leakage.^26^ Owing to the relatively high conductivity of the ferromagnetic (or ferrimagnetic) phase that forms the nanopillars through the film thickness of the VANs, the measurement of CME effect by applying a large electric field is challenging. However, scanning probe microscopy has been often used to characterize ME effect to avoid the leakage issue. For example, Zavaliche et al. reported electric field–controlled magnetization switching in BiFeO_3_:CoFe_2_O_4_ VANs, characterized by both piezoresponse force microscopy (PFM) and magnetic force microscopy (MFM).^28^, ^29^ In fact, PFM has become an alternative way to measure DME effect under different magnetic fields as reported in core–shell nanofibers and nanoparticle‐doped films.^30^, ^31^

The ME couplings have been intensively studied in layered heterostructures with lateral interfaces. The discovered mechanisms such as interfacial strain, charge effect, or exchange bias effects have been widely used to design a variety of devices.^4^ However, the application of its counterpart, VANs with vertical interfaces, has been hindered by the lack of better understanding of the coupling mechanisms. For example, what are the roles of vertical interfacial area and pillar size in the ME couplings in VANs? Vertical interfacial area, directly controlled by pillar size, is one of the most fundamental parameters in VANs as it affects vertical strain as well as functionalities.^10^ In this work, we have investigated the multiferroic properties and ME coupling mechanisms in BTO:CFO VANs. With a magnetic field applied along the out‐of‐plane (*z*‐axis) direction, magnetic field–dependent second harmonic generation (SHG) demonstrates the longitudinal ME coupling. With a magnetic field applied along the in‐plane (*x–y*‐axis) direction, the transverse ME coupling strength can be estimated by using PFM. Large ME coupling coefficients in BTO:CFO VANs were estimated from the magnetic field–dependent PFM. The possible underlying mechanisms governing the coupling between ferroelectric (FE) and ferrimagnetic or ferromagnetic (FM) phases in VANs with these two configurations were further studied by using phase field simulation with different pillar sizes. Phase field simulations reveal that the pillar size–dependent ME coupling is owing to the competition between the vertically coupled lattice (interface effect) and the bulk volume conservation effect (bulk effect). The impact of the discovered ME coupling mechanisms in VANs is beyond the material systems and properties studied here, and it can be applied to tune functional properties in many other VANs and related devices which utilize vertical interface coupling effects.

Vertically aligned epitaxial BTO:CFO nanocomposites with CFO ferrimagnetic nanopillars embedded in BTO ferroelectric matrix were synthesized by pulsed laser deposition. Both SrTiO_3_ (STO) (001) and Nb:STO were used as the substrates. The structural properties such as the epitaxial relationship and the crystallinity of the nanocomposites are evaluated by X‐ray diffraction (XRD) 2θ–ω and in‐plane *Φ* scans (Figure S1, Supporting Information). **Figure** **1**a shows the reciprocal space mapping (RSM) of a 720 nm thick nanocomposite film. In such a nanocomposite film, the lattice strain is mainly controlled by the vertical interface between BTO and CFO phases. The BTO matrix is in tension (+0.38%) and the CFO nanopillars are under compression (−1.4%) along the vertical direction. Figure 1b shows an out‐of‐plane magnetic anisotropy in the CFO nanopillars. The magnetic anisotropy is dominated by the large vertical compressive strain in CFO nanopillars, although the shape anisotropy also plays a role.^32^, ^33^ To explore the magnetic anisotropy, the angle‐dependent magnetization was measured with a fixed external magnetic field,^34^ and the measurement setup was detailed in the Supporting Information. As shown in Figure 1c, the switch of transverse magnetization at 90° (270°) indicates the uniaxial switching of the out‐of‐plane magnetic moments (Figure S2, Supporting Information). The atomic force microscopy (AFM) image in Figure 1d shows that CFO nanopillars are uniformly embedded in the BTO matrix. The inset of Figure 1d shows a 3D schematic diagram of the nanocomposite structure with vertical nanopillars embedded in a film matrix. To study the microstructure of these nanocomposites, scanning transmission electron microscopy (STEM) under the high‐angle annular dark‐field (HAADF) mode was conducted. This plan‐view STEM image clearly shows a well‐defined distribution of CFO nanopillars in the BTO matrix with an average nanopillar feature size of ≈20 nm (Figure 1e). Antiphase boundaries (APBs), marked in white arrows (Figure 1e), are formed to relax the lattice mismatch along the radial direction which has also been reported in other systems.^35^ An atomic‐resolution HAADF–STEM image seen from the plan‐view direction (Figure 1f) shows the boundary between CFO and BTO along the lateral interface. It is noted that the interface is sharp.

**Figure 1:**
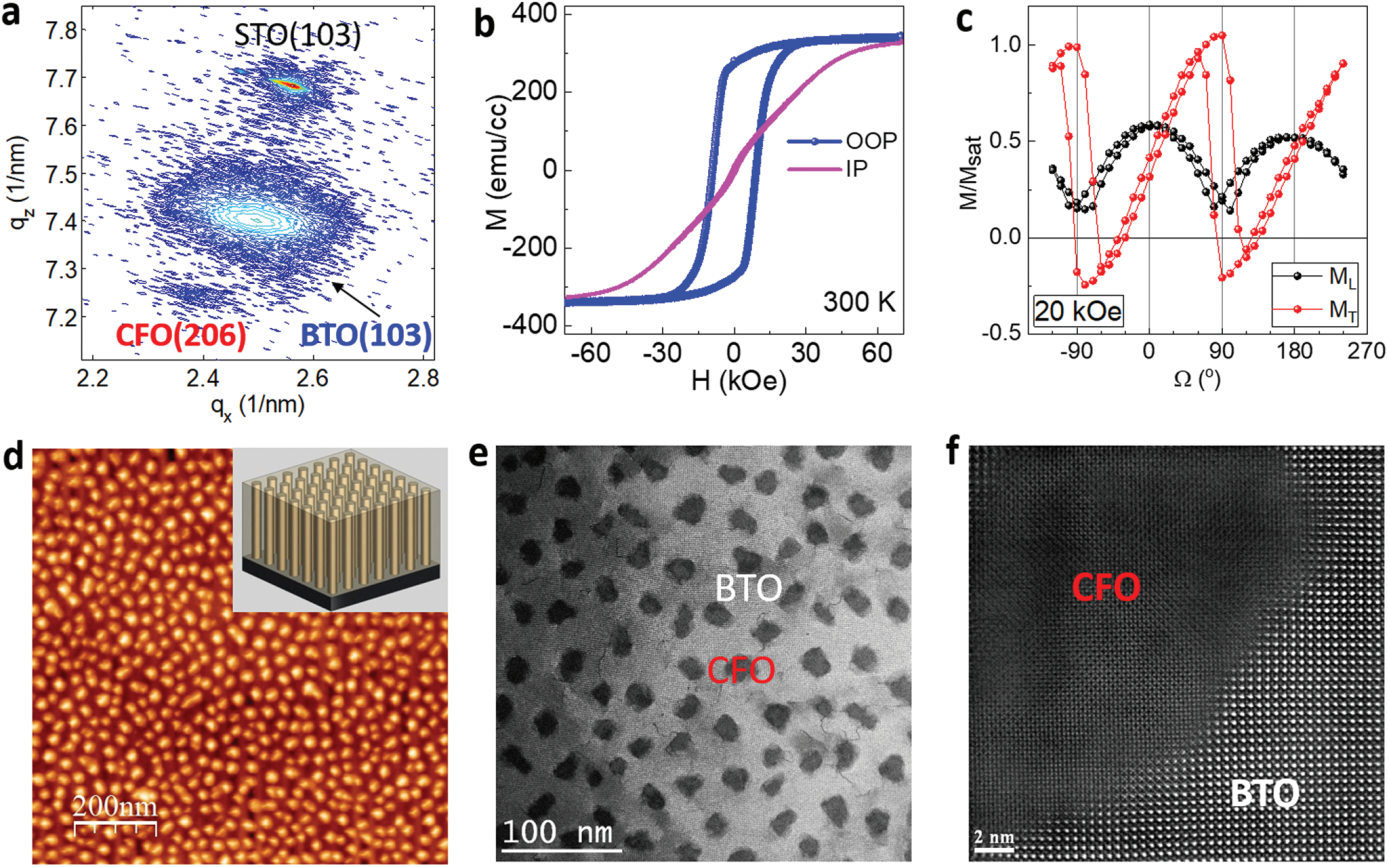
Structural and microstructure of BTO:CFO VANs. a) RSM of BTO:CFO nanocomposite films on STO substrates around the STO (103) region. b) In‐plane and out‐of‐plane magnetization at 300 K. c) Angle‐dependent magnetization for longitudinal (*L*) and transverse (*T*) magnetic components under an external magnetic field of 20 kOe. d) AFM image of BTO:CFO nanocomposites. e) Plan‐view STEM image showing CFO nanopillars in BTO matrix. The dark region represents the CFO phase. The gray area shows the BTO matrix. f) Atomic‐resolution HAADF–STEM image showing the interface between BTO and CFO.

Since SHG is a sensitive probe of broken inversion symmetry, this technique can be directly used to probe ferroelectric order,^36^, ^37^, ^38^ which breaks inversion symmetry for bulk crystals. In our SHG setup, the magnetic field (*H_z_*) was applied along the out‐of‐plane (*z*‐axis) direction. In our experiment, we measured the SHG signals at different temperatures for analyzers oriented along 90° (S‐out) and 0° (P‐out) (Figure S4, Supporting Information). This method has been described in detail in the previous work.^38^, ^39^ In order to accurately track the temperature dependence of the SHG signal, we take the amplitude of the P‐out SHG signal for 0° incident polarization (P‐in) and plot it as a function of temperature, as shown in **Figure** **2**a. A sharp increase in the SHG intensity can be observed as the temperature is decreased below 330 K, which is associated with the FE transition in BTO. The relatively weak SHG signal above the transition temperature arises from the surface of the sample or the interface between the BTO and the CFO where the inversion symmetry is broken. The large change of SHG intensity at 280–300 K can be attributed to the tetragonal–orthorhombic transition. The gradual increase of SHG intensity at 300–400 K region might indicate a spatially inhomogeneous phase transition in BTO matrix which is due to nonuniform strain distribution. Figure 2b,c shows the SHG signal measured at 295 K for different azimuthal angles while rotating the sample along the *z‐*axis for P‐out and S‐out, respectively. Neither the symmetry nor the intensity of the SHG signal changes with azimuthal angles, suggesting that the ferroelectric polarization in BTO is oriented along the *z‐*axis. Therefore, both magnetization and polarization were found to be aligned along the out‐of‐plane direction (*z*‐axis) due to the out‐of‐plane tensile strain in the BTO phase and compressive strain in the CFO phase.

**Figure 2:**
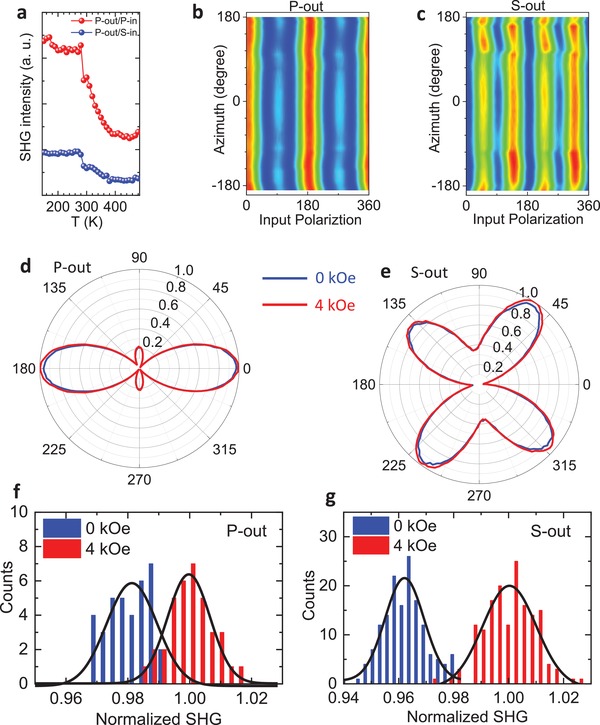
a) Temperature dependence of the SHG intensity for P‐out polarization. b,c) The SHG intensity (295 K) at different azimuth angles while rotating the sample along the *z*‐axis for P‐out and S‐out polarizations, respectively. d,e) Comparison of the SHG signals (295 K) at 4 kOe (red curve) and zero field (blue curve) for P‐out and S‐out polarizations, respectively. The magnetic field is applied along the *z*‐axis. f,g) Histogram of the SHG intensity (295 K) at 4 kOe (red) and zero field (blue) for P‐out and S‐out polarizations, respectively.

To demonstrate the ME coupling in BTO:CFO VANs, we compare the SHG signals along the *z‐*axis for a magnetic field of 4 kOe (the highest in this setup) to zero field data. The blue (red) curves in Figure 2d,e show polar plots of the SHG signal at *H_z_* = 0 and *H_z_* = 4 kOe for P‐out and S‐out polarizations, respectively. It is evident that for both output polarizations, the SHG intensity is enhanced by applying a 4 kOe magnetic field along the *z‐*axis. The enhanced polarization under a magnetic field is a clear indication of the ME coupling between the CFO and BTO. The field‐induced enhancement is ≈3%. To conclusively verify this, we made a large number of scans with and without the magnetic field and also performed a statistical analysis to lend further credence to the existence of ME coupling. Figure 2f,g depicts histograms of the SHG intensity over many scans at 4 kOe (red) and zero field (blue) for P‐out and S‐out, respectively. For both polarizations, the counts at different intensities are clearly separated into two groups, further demonstrating the existence of ME coupling in the BTO:CFO nanocomposites. By comparing the average of the SHG intensity from a large number of scans for both polarizations, a 3% enhancement of the SHG signal at a 4 kOe magnetic field was obtained, which indicates strong ME coupling in BTO:CFO VANs.

To evaluate the ME coupling strength in BTO:CFO VANs, we have studied the switching behaviors under different magnetic fields by PFM with the magnetic field parallel to the in‐plane direction,^30^, ^40^ where the magnetic field is termed as *H_x_*. The transverse ME coupling coefficient (α_31_) can be estimated. **Figure** **3**a shows a typical corrected PFM amplitude image of the BTO:CFO nanocomposites. The blue area represents the non‐ferroelectric CFO nanopillars and the other areas represent the piezoelectric BTO matrix, where DC fields have been applied to switch the polarization in the VAN thin films. Figures 3b shows the corresponding butterfly loop due to the piezoresponse, and the PFM phase hysteresis loop was shown in Figure S5 (Supporting Information). It can be seen that the maximum PFM amplitude (with an *H_x_* of 2 kOe) at the saturated electric fields drops, and the corresponding coercive voltage increases. The increased coercive field indicates that it is harder to switch ferroelectric domains due to the coupling between the BTO matrix and the CFO nanopillars. Similar effects have been reported in core–shell nanostructures.^30^ The phase contrast curves of the switching (Figure S5, Supporting Information) are close to 180°, indicating complete polarization switching.

**Figure 3:**
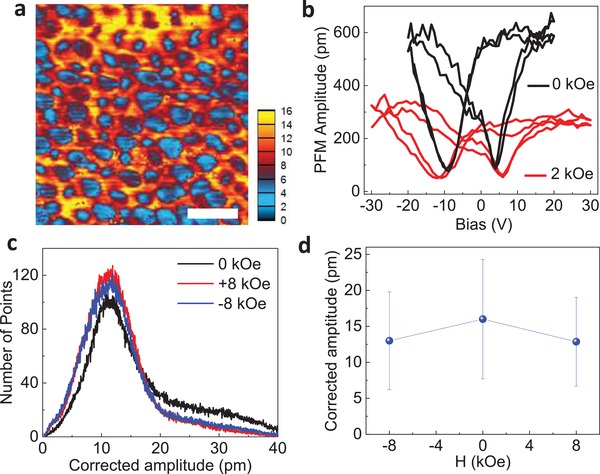
a) Corrected PFM amplitude mapping of the BTO:CFO nanocomposite film at a 2 kOe magnetic field. The scale bar in panel (a) is 100 nm. b) Amplitude–voltage butterfly loop of BTO:CFO nanocomposite films before and after the application of an external magnetic field of 2 kOe by using a variable field module. c) Amplitude histogram under different magnetic fields. d) The corrected PFM amplitude under different magnetic fields (0 and ±8 kOe).

The transverse ME coefficient α_31_ of BTO:CFO VANs has been calculated using the following approximation:^30^α_31_ = Δ*E*_3_/Δ*H*_1_, where Δ*E*_3_ is the change in longitudinal electric field induced by the change in lateral magnetic field Δ*H*_1_. The piezoelectric constant *d*_33_ of the BTO phase can be estimated as ≈30 pm V^−1^ from the amplitude butterfly curve at zero magnetic field. The change in electric field is given by Δ*E*_3_ = Δ*u*/*d*_33_ *h*, where Δ*u* is the change in piezoresponse displacement and *h* is the film thickness (320 nm). To estimate the ME coefficient, we have mapped the BTO matrix statistically. The statistical distribution of the corrected PFM amplitude is shown in Figure 3c. It is clear that the PFM amplitude is almost the same at fields of ±8 kOe, indicating the reliability of PFM measurements. The PFM amplitude distribution features a peak at ≈11 nm and a long tail. The peak could be assigned to regions near to the vertical interface, and the long tail with higher PFM response could be assigned to regions far away from the interface. The change of the PFM amplitude distribution in magnetic fields suggests that BTO regions away from the interface dominates the ME coupling. Figure 3d shows the average PFM amplitude under different applied fields by integrating the data in Figure 3c. It shows that the PFM amplitude is reduced by applying an external magnetic field, which is consistent with the magnetic field–dependent butterfly loop shown in Figure 3b. The ME coefficient α_31_ was estimated to be ≈390 mV cm^−1^ Oe^−1^ at 8 kOe, which is higher than most reported values (10–100 mV cm^−1^ Oe^−1^),^16^, ^17^, ^18^, ^19^, ^20^ but is smaller than the theoretical prediction (≈2000 mV cm^−1^ Oe^−1^).^14^ As discussed later, pillar size could be used to further optimize the coupling strength.

In ferromagnetic/ferroelectric bilayers or heterostructures, strain, charge, and exchange bias effects contribute to the ME couplings.^24^ In order to understand the ME coupling mechanisms in VANs, we carried out phase field simulations to explain the magnetic field direction–dependent piezoelectric response of the BTO matrix as observed in SHG and PFM measurements. **Figure** **4**a shows the configuration of the external magnetic field *H_z_* parallel to the CFO nanopillars, the same as the SHG configuration. Figure 4b shows the distribution of the calculated out‐of‐plane strain change, *Δε*_33_, in the *x–y* plane of the BTO matrix with an external magnetic field *H_z_*. As shown from the data, the *Δε*_33_ of the BTO matrix is significantly modified by the *H_z_*. Interestingly, the BTO matrix surrounding the CFO nanopillar is not uniformly strained due to ferroelectric domain structure. The average *Δε*_33_ is positive with applying *H_z_* along the out‐of‐plane direction which is consistent with the SHG results. We speculate the following processes happened after applying the external magnetic fields *H_z_*. The CFO pillars shrink along the *z*‐axis due to the negative magnetostrictive coefficient. The CFO pillars also expand along the *x–y* (in‐plane) direction. This will induce two effects. On the one hand, the shrinkage of the CFO pillars along the *z*‐axis tends to compress the adjacent BTO matrix due to the coupled lattice at the vertical interfaces (called interface effect). On the other hand, the in‐plane expansion of the CFO pillars squeezes the surrounding BTO matrix (volume conservation of the VAN system) in the *x–y* plane which makes the BTO matrix in tension along the *z*‐axis due to the bulk volume conservation of the BTO matrix. A similar volume conservation effect has been used to explain cooling induced strain in VANs.^41^ Such a competing effect has also been discussed by Schmitz‐Antoniak et al.^15^ For simplicity, the volume conservation is defined as a bulk effect and the vertical lattice coupling is defined as an interface effect. An illustration in Figure S6 (Supporting Information) describes a simple picture of the competing interface and bulk effects. The total SHG signal in the BTO:CFO VANs is enhanced with *H_z_*, indicating the domination of the bulk volume conservation effect in this studied sample.

**Figure 4:**
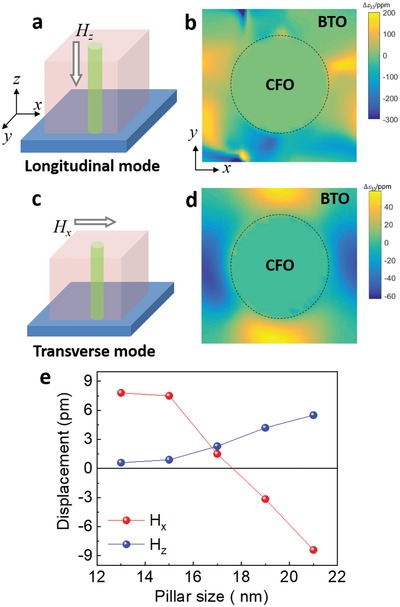
a) An illustration of the longitudinal ME coupling mode with the magnetic field (*H_z_*) parallel to the CFO nanopillars. b) The calculated distribution of the out‐of‐plane strain change, *Δε*_33_, in the *x–y* plane of BTO with an external magnetic field *H_z_*. The dashed black circle represents a CFO pillar. ppm represents 10^−6^. An *H_z_* of 0.5 kOe is used in simulation. c) An illustration of the transverse ME coupling mode with the magnetic field parallel to film surface (*H_x_*). d) The calculated *Δε*_33_ distribution in the *x–y* plane of BTO with an external magnetic field *H_x_*. An *H_x_* of 4.4 kOe is used in simulation. The pillar size used in panels (b) and (d) is 21 nm. e) CFO nanopillar size–dependent macroscopic displacement of the BTO matrix in the presence of external magnetic fields *H_x_* and *H_z_*.

Figure 4c shows the configuration of the external magnetic field *H_x_* perpendicular to the CFO nanopillars, the same as the PFM setup. As shown in Figure 4d, the out‐of‐plane strain change, *Δε*_33_, is positive along the *y*‐axis and negative along the *x*‐axis. Similar competing effects exist in the *H_x_* configuration. The CFO pillars are compressed along the *x‐*axis and elongated along both the *z*‐axis and the *y*‐axis. On the one hand, the CFO pillars along the *z*‐axis will elongate the adjacent BTO matrix along the *z*‐axis (interfacial effect). On the other hand, the shrinkage of the CFO pillars along the *x*‐axis leads to the expansion of the BTO matrix along the *x*‐axis (bulk effect). Such an effect tends to reduce the out‐of‐plane lattice of the BTO matrix. Apparently, these factors have “opposite” effects on the ME coupling. The average PFM amplitude of the BTO matrix was suppressed, indicating the domination of the bulk effect. This result is consistent with the change of PFM amplitude distribution as a function of *H_x_* (Figure 3c,d) that ME coupling is dominated by BTO regions away from the vertical interface. Although the measurement configurations in SHG (*H_z_* configuration) and PFM (*H_x_* configuration) are completely different, the conclusions are consistent with each other. Both results confirm that the ME coupling in the studied sample (pillar size of ≈20 nm) is controlled by the bulk effect rather than the interfacial effect.

Since the ME coupling in VANs is controlled by the competition of bulk and interface effects, it should change with the pillar diameter (with a fixed volume ratio) or, in other words, the vertical interface density. Phase field simulation results in Figure 4e show the pillar diameter–dependent out‐of‐plane displacement in BTO. With the magnetic field *H_z_* parallel with the pillars, the displacement of BTO is positive and increases with the pillar size (blue curve in Figure 4e) from 12 nm to 22 nm. This is reasonable because VANs with larger CFO pillar size and thus smaller vertical interface density exhibit less interface effect. It is no wonder that our VANs (CFO pillar size of ≈20 nm) exhibit the bulk volume–dominated ME coupling in the *H_z_* configuration. Interestingly, the macroscopic displacement of BTO decreases from positive to negative with increasing the CFO pillar size in the *H_x_* configuration. When the CFO pillar size is small (<17 nm), the vertical interface density is high which favors the interface controlled ME couplings. Therefore, the BTO displacement is positive as discussed above. Upon increasing the CFO pillar size, the vertical interface density decreases and the bulk volume effect takes over. Therefore, the BTO displacement changes to negative. Although the pillar size–dependent ME coupling simulation is not validated by experimental results, our work reveals the origin of the underlying coupling mechanisms in VANs and provides guidance to design and tune functionalities of VANs in general via changing the vertical interfacial area which is ultimately controlled by pillar size.

Epitaxial BTO:CFO VANs with CFO nanopillars in a BTO matrix show strain coupling along the vertical interface between CFO and BTO phases. Such a vertical strain produces both polarization and magnetization aligned along the out‐of‐plane direction. Longitudinal and transverse ME couplings were studied based on SHG and PFM measurements, respectively. Phase field simulations reveal that the ME coupling in VANs is controlled by the competition between vertical interface and the bulk volume conservation effect which strongly depends on the nanopillar size or the vertical interface density. Our work explores the ME coupling in VANs and provides a pathway to design a variety of multiferroic VAN thin films with large ME coupling strength for potential device applications.

## Conflict of Interest

The authors declare no conflict of interest.

## Supporting information

Supplementary
