# Supplementary material for: Competing Interface and Bulk Effect–Driven Magnetoelectric Coupling in Vertically Aligned Nanocomposites
Source: Adv Sci (Weinh). 2019 Aug 2;6(19):1901000. doi: 10.1002/advs.201901000 (PMC6774036; doi:10.1002/advs.201901000)
Supplement: Supplementary file 1 — Supplementary [file ADVS-6-1901000-s001.pdf]

Copyright WILEY-VCH Verlag GmbH & Co. KGaA, 69469 Weinheim, Germany, 2019.

ADVANCED SCIENCE
Open Access

Supporting Information

for *Adv. Sci.,* DOI: 10.1002/advs.201901000

Competing Interface and Bulk Effect–Driven Magnetoelectric Coupling in Vertically Aligned Nanocomposites

Aiping Chen, Yaomin Dai, Ahmad Eshghinejad, Zhen Liu, Zhongchang Wang, John Bowlan, Erik Knall, Leonardo Civale, Judith L. MacManus-Driscoll, Antoinette J. Taylor, Rohit P. Prasankumar, Turab Lookman, Jiangyu Li, Dmitry Yarotski, and Quanxi Jia**

Supporting information

Methods

Thin film growth. Pulsed laser deposition (PLD, KrF excimer laser, λ = 248 nm) was employed to grow BTO:CFO films on $SrTiO_3$ (STO) (001), $SrRuO_3$ (SRO) buffered STO and Nb:STO. BTO:CFO VAN films was grown by using a densely pressed and uniformly mixed BTO:CFO ceramic target with a molar ratio of 65:35. During the thin film growth, the substrate temperature was maintained at 725 to 900 °C. Films with different thicknesses (200 nm to 720 nm) were deposited under 50 mTorr oxygen pressure and 10 Hz laser frequency. Laser energy of 2.0 J/cm^2 was used to grow these VAN films. To get uniform laser energy density, a rectangular shaped laser beam, defined by the image beam technique, was used for the film growth. After deposition, the films were *in situ* annealed at 600 °C and 1 atm oxygen for 1 h before cooling down to room temperature at 5 ^oC/min.

SHG measurements: Our SHG measurements were carried out using an amplified Ti:Sapphire laser system, which produces pulses with a center wave length of 780 nm (1.59 eV), a duration of ~100 fs, and a repetition rate of 250 kHz. Using a 1.59 eV probe beam, a SHG signal of 3.18 eV is generated from the BTO:CFO VAN films. The SHG intensity is detected by a photomultiplier tube with lock-in detection after filtering out the 1.59 eV beam. The polarization of the incident light is controlled by a half-wave plate, and the SHG signal in the reflection from the sample with an angle of incidence at 45° is measured for either P-out or S-out polarizations. For both polarizations, data was collected at different temperatures from 200 to 480 K. At T = 295 K, the SHG intensity was measured at different azimuth angles while rotating the sample along the z axis. In addition, we measured the SHG signal at T = 295 K in a magnetic field of 4

kOe along the *z* axis.

Magnetic characterization: The magnetization *vs.* magnetic field (*M-H*) loops were systematically investigated by the vibrating sample magnetometer option in Physical Properties Measurement System (PPMS, Quantum Design). The in-plane and out-of-plane *M-H* loops were measured by applied the field parallel and perpendicular to the film plane during magnetic measurements. The angular dependent magnetization measurements were performed in a Quantum Design MPMS SQUID magnetometer, equipped with a rotating sample holder and two sets of pickup coils that allowed measurement of the longitudinal (M_l) and transverse (M_t) components of **M** (||**H** and ⊥**H** respectively). We developed a simple model to simulate the angular dependent magnetization of the sample (see Fig. S2). It describes the magnetization vector **M** as the linear superposition of two components, the out-of-plane $\mathbf{M}_{oop}$ (normal to the film surface) and the in-plane $\mathbf{M}_{ip}$, (parallel to the surface), $\mathbf{M} = \mathbf{M}_{oop} + \mathbf{M}_{ip}$, see Fig. S2a. The model assumes that $\mathbf{M}_{oop}$ depends only on the component of the applied field **H** normal to the film surface, $\mathbf{H}_{\perp}$ (parallel to the pillars), while $\mathbf{M}_{ip}$ depends only on the component of **H** parallel to the surface, $\mathbf{H}_{||}$. To simulate the sample response shown in Fig. 1b, the model assumes that $\mathbf{M}_{oop}$ is purely ferromagnetic (hysteretic) and $\mathbf{M}_{ip}$ is linear paramagnetic, as shown in Fig. S2b. As the sample rotates at fixed **H**, it is exposed to variable fields $H_{\perp} = Hcos(\Omega)$ and $H_{||} = Hsin(\Omega)$. The resulting longitudinal and transverse signals $M_L(\Omega)$ and $M_T(\Omega)$ are shown in Fig S2c, where the curves labeled 1 and 2 indicate clockwise and anticlockwise rotations, respectively. This simple model qualitatively reproduces all the main features of Fig. 1c.

PFM measurements: The ferroelectric properties of nanocomposite thin films were examined by PFM using an Asylum Research MFP-3D atomic force microscope (AFM). To characterize ME coupling of nanocomposites, an Asylum Research Variable Field Module (VFM) was used to apply magnetic fields parallel to the film plane during PFM characterization to examine the switching characteristics induced by the external magnetic field, from which lateral (transverse)

ME coefficient can be estimated. The topography image was used as a marker to ensure that PFM images from the same area are compared under different magnetic fields.

Modeling and phase field simulation: In the nanocomposite with ferromagnetic pillars embedded in the ferroelectric matrix, the connection between polarization **P** (P_1, P_2, P_3) and magnetization **M** (m_1, m_2, m_3) can be obtained from the minimization of the free energy,

$$\mathrm{F} = (1-\alpha)\left(G_p(\mathbf{P}) - \mathbf{EP}\right) + \alpha(G_m(\mathbf{M}) - \mathbf{HM}) + G_{ela} \qquad (1)$$

where α is the fraction of the CFO pillars in the nanocomposite, and **E** and **H** represent the applied electric field and magnetic field. $G_p(\mathbf{P})$ and $G_m(\mathbf{M})$ are the free energy of ferroelectric matrix and ferromagnetic pillars.[1, 2] The last term in Eq. (1) denotes the elastic energy. In the system, the total strain ε_{ij} can be obtained by solving the elastic equilibrium equation$\frac{\partial[c_{ijkl}(\varepsilon_{kl}-\varepsilon^0_{kl})]}{\partial x_j} = 0$, where the spontaneous strain ε^0_{ij} can be written as,[3]

$$\varepsilon^0_{ij} = \begin{cases} \alpha\left[\frac{3}{2}\lambda_{100}\left(m_i m_j - \frac{1}{3}\right)\right] + (1-\alpha)Q_{ijkl}P_kP_l\,, & (i = j) \\ \alpha\left[\frac{3}{2}\lambda_{111}(m_i m_j)\right] + (1-\alpha)Q_{ijkl}P_kP_l\,, & (i \neq j) \end{cases} \qquad (2)$$

Where Q_{11} = 0.11 $C^{-2}m^4$, Q_{12} = -0.045 $C^{-2}m^4$ and Q_{44} = 0.03 $C^{-2}m^4$ are electrostrictive coefficients of BTO, and $\lambda_{100} = -590 \times 10^{-6}$ and $\lambda_{111} = 120 \times 10^{-6}$ are saturation magnetostriction of CFO. For simplicity, we used the same elastic elastic constants for BTO and CFO, i.e. C_{11} = 17.8×10^{10} N/m^2, C_{12} = 9.6×10^{10} N/m^2, and C_{44} = 12.2×10^{10} N/m^2. The lattice parameters of BTO and CFO are 4 Å and 8.38 Å, respectively. Due to the out of plane misfit, the CFO pillar is compressed and BTO matrix is elongated along the axis direction in order to reduce the elastic energy, the total elastic energy of the system can be finally written as:

$$G_{ela} = -\frac{1}{2}\int\frac{d^3\mathbf{k}}{2\pi^3}\{n_i\sigma^0_{ij}(\mathbf{k})\Omega_{jl}[\sigma^0_{lm}(\mathbf{k})]^* n_m\} + \frac{1}{2}\phi(S_1, S_2, \alpha)(\Delta\varepsilon)^2 \qquad (3)$$

Where $\sigma_{ij}^0 = C_{ijkl}\varepsilon_{kl}^0$, $\mathbf{k}$ is the wave vector in the reciprocal space and $n_i = \frac{k_i}{|\mathbf{k}|}$ is the unit vector. The tensor $\Omega_{ik}{}^{-1} = C_{ijkl}n_j n_l$. The second term in Eq. (3) denotes the elastic interaction between CFO pillars and BTO matrix due to the out of plane misfit.[4] $\phi(S_1, S_2, \alpha)$ is an elastic coefficient in dependent on CFO fraction α and the elastic compliance S. $\Delta\varepsilon = \Delta\varepsilon_T + \bar{\varepsilon}_{33}^{p}(P) + \bar{\varepsilon}_{33}^{M}(M)$, where $\Delta\varepsilon_T$ is the misfit at deposition, $\bar{\varepsilon}_{33}^{p}(P)$ and $\bar{\varepsilon}_{33}^{M}(M)$ are the average spontaneous ferroelectric stain and ferromagnetic strain along the out of plane direction. In the phase field simulation, the temporal evolution of the polarization in BTO and the magnetization in CFO is simulated by solving the time-dependent Ginzburg Landau equation[1] and the Landau–Lifshitz–Gilbert equation.[2] Periodic boundary condition is used to simulate CFO pillar arrays embedded in the BTO matrix. The parameters we used in the simulation can be found in Ref [3]. The pillar size used in Figure 4 simulation is 21 nm.

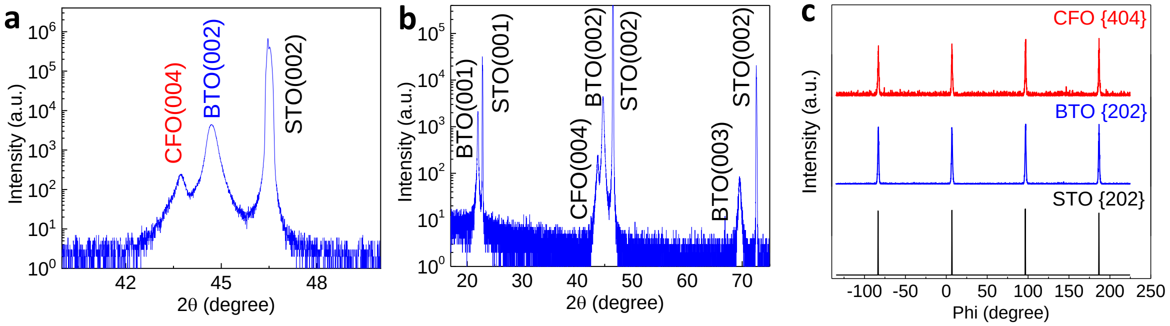

Figure S1. XRD of typical BTO:CFO nanocomposite films on STO (001) substrates (a) local scan and (b) full scan. (c) Phi scans of CFO, BTO and STO. Figure S1a is a typical XRD 2θ-ω scan of BTO:CFO (65:35) nanocomposite films at 850 ^{o}C. Both BTO and CFO phases are textured along [001] direction out-of-plane. The Φ scan confirms the in-plane texture and the epitaxial relationship is BTO (002) // CFO (004) //STO (002) and BTO [200] // CFO [400] //STO [200]. The results reveal the cubic on cubic growth.

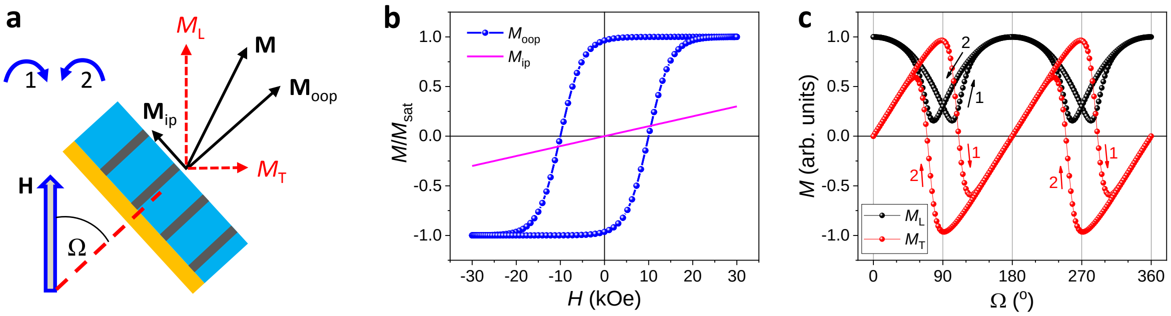

Figure S2. (a) The configuration of the angle dependent magnetization measured with the external magnetic field along the longitudinal direction. (b) The simulated out-of-plane and in-plane *MH* loops for CFO pillars. (c) The simulated angle dependent magnetization with Longitudinal (L) and Transverse (T) magnetic components under an external magnetic field of 30 kOe. Such simulated curves are similar with Figure 1c measured in 20 kOe. The arrows 1 and 2 in (a) are corresponding to 1 and 2 in (c).

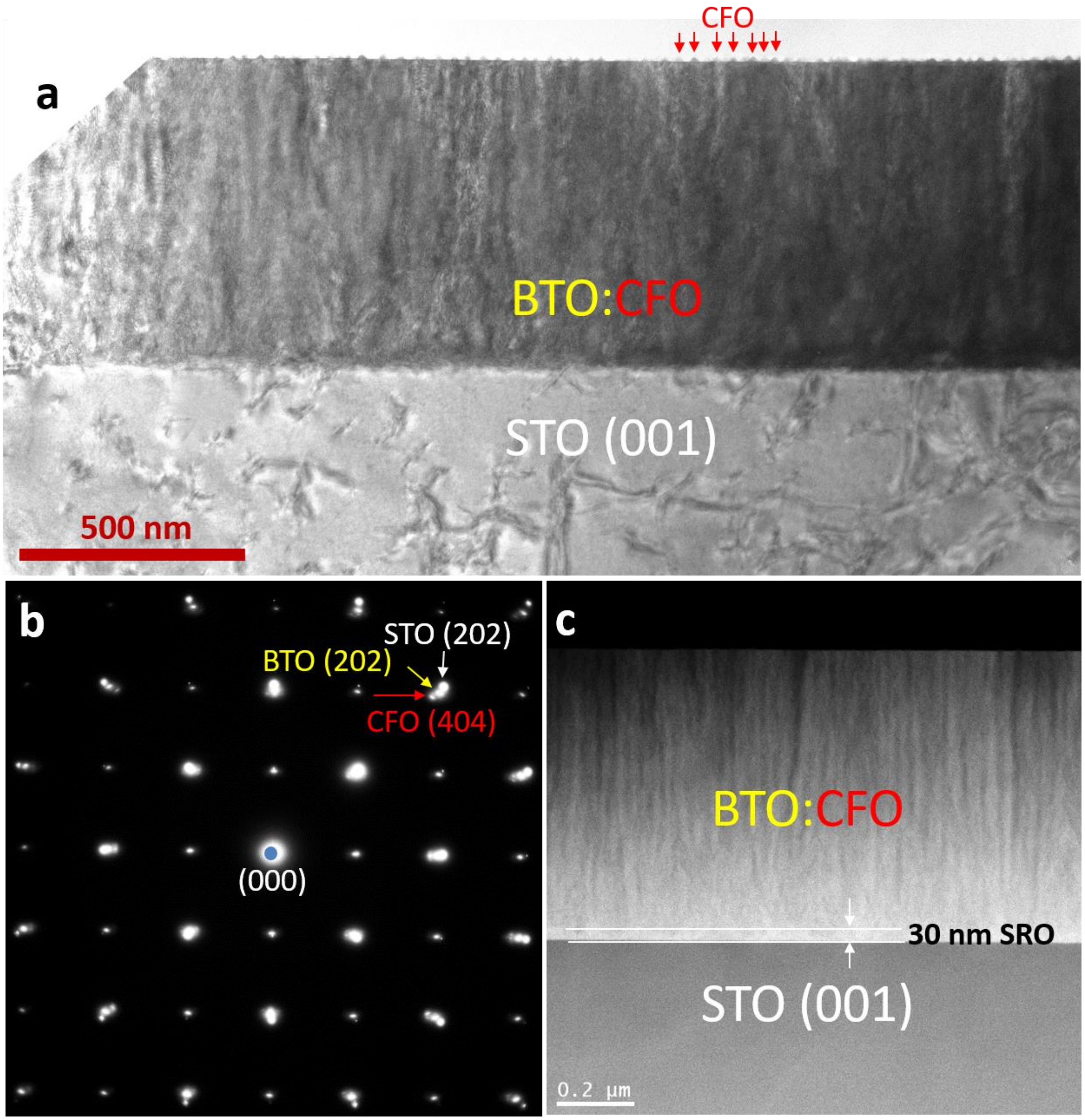

Figure S3. Microstructure of BTO:CFO VAN thin films. (a) Cross sectional low magnification TEM image of the 720 nm BTO:CFO VAN thin films on STO (001). (b) SAED pattern of such a VAN film. (c) Low magnification STEM image of the BTO:CFO VAN film with a 30 nm SRO bottom electrode layer.

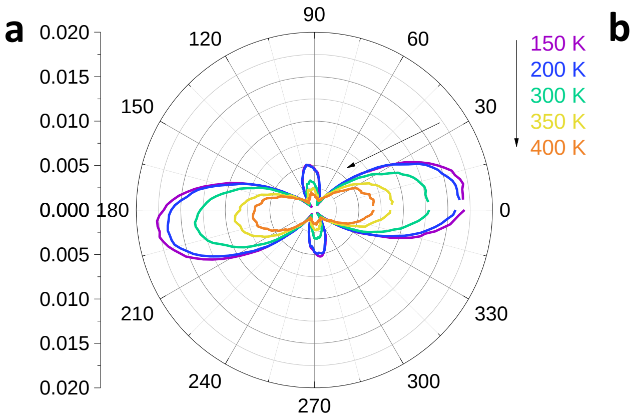

Figure S4. Temperature-dependent SHG signal for analyzers along (a) 90 (S-out) and (b) 0 (P-out) degrees.

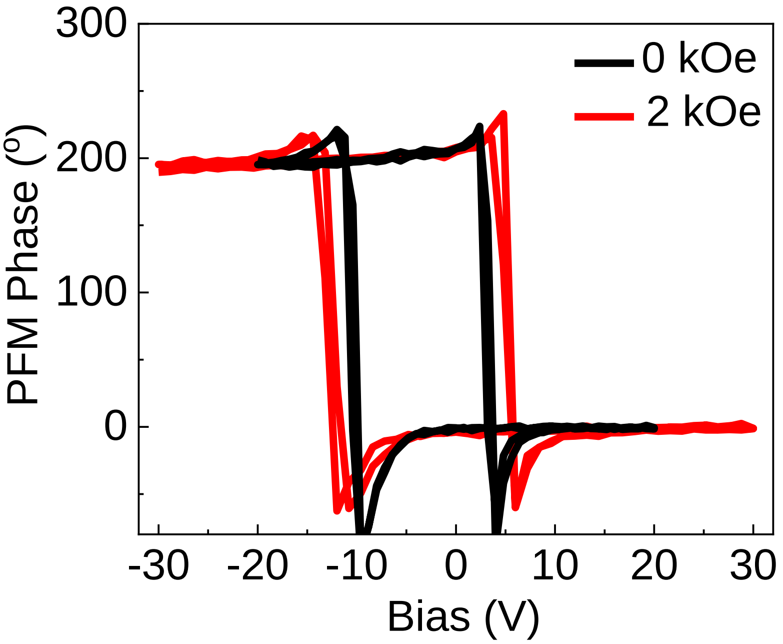

Figure S5. Phase-voltage hysteresis loop of BTO:CFO nanocomposite films before and after the application of external magnetic field of 2 kOe by using a Variable Field Module.

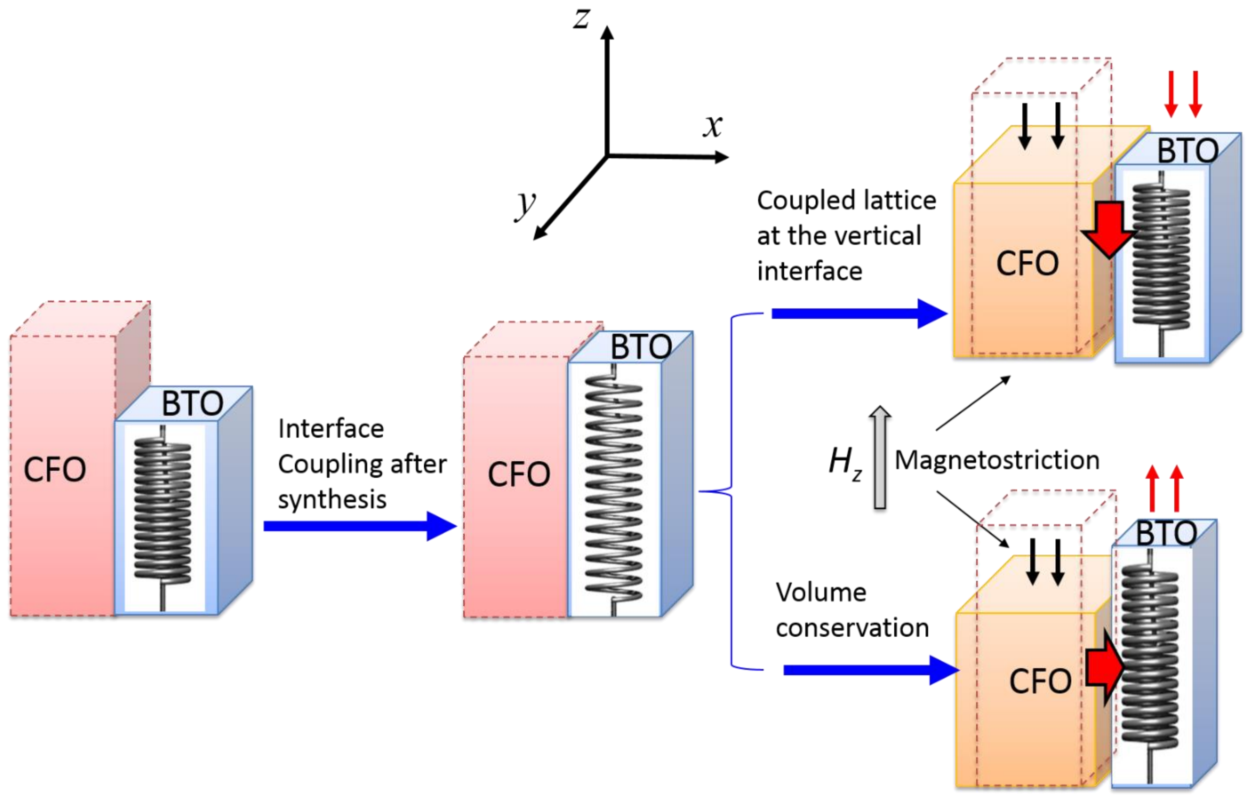

Figure S6. An illustration of the competing effect in the case of H_z. After applying the H_z, the CFO nanopillars shrink along the *z*-axis direction and expanse along the *x*-*y* directions due to the negative magnetostriction of CFO. The lattice coupling at the vertical interface tends to lower the adjacent BTO matrix (as marked by the red arrow). On the other hand, the expansion of CFO in-plane will induce the in-plane shrinkage of the BTO matrix (as marked by the red arrow), as required by the bulk volume conservation effect. Then, the BTO matrix will be elongated along the *z*-axis. For simplicity, we used cuboids to illustrate the CFO pillars and the BTO matrix.

Reference

1. Hu, H. L., Chen, L. Q. Three-dimensional computer simulation of ferroelectric domain formation. *J. Am. Ceram. Soc.* **81**, 492-500 (1998).

2. Zhang, J., Chen, L. Phase-field microelasticity theory and micromagnetic simulations of domain structures in giant magnetostrictive materials. *Acta Mater.* **53**, 2845-2855 (2005).

3. Wu, P., Ma, X., Zhang, J., Chen, L. Phase-field model of multiferroic composites: Domain structures of ferroelectric particles embedded in a ferromagnetic matrix. *Philos. Mag.* **90**, 125-140 (2010).

4. Zheng, H.*, et al.* Multiferroic $BaTiO_3$-$CoFe_2O_4$ nanostructures. *Science* **303**, 661-663 (2004).
